# Fungus-Mediated Preferential Bioleaching of Waste Material Such as Fly - Ash as a Means of Producing Extracellular, Protein Capped, Fluorescent and Water Soluble Silica Nanoparticles

**DOI:** 10.1371/journal.pone.0107597

**Published:** 2014-09-22

**Authors:** Shadab Ali Khan, Imran Uddin, Sana Moeez, Absar Ahmad

**Affiliations:** Division of Biochemical Sciences, CSIR- National Chemical Laboratory Pashan, Pune, India; King Abdullah University of Science and Technology, Saudi Arabia

## Abstract

In this paper, we for the first time show the ability of the mesophilic fungus *Fusarium oxysporum* in the bioleaching of waste material such as Fly-ash for the extracellular production of highly crystalline and highly stable, protein capped, fluorescent and water soluble silica nanoparticles at ambient conditions. When the fungus *Fusarium oxysporum* is exposed to Fly-ash, it is capable of selectively leaching out silica nanoparticles of quasi-spherical morphology within 24 h of reaction. These silica nanoparticles have been completely characterized by UV-vis spectroscopy, Photoluminescence (PL), Transmission electron microscopy (TEM), X-ray diffraction (XRD), Fourier transform infrared spectroscopy (FTIR) and Energy dispersive analysis of X-rays (EDAX).

## Introduction

Fly- ash is the finest coal combustion product obtained after the burning of pulverized coal to generate power. It consists of small particles of inorganic minerals, with some carbon. It is almost spherical in shape which gives it characteristic flowing ability. Because of the high content of silica, it normally exhibits pozzolanic and sometimes cementitious behavior. It is therefore an economical additive in cement and can also be used to stabilize loose soils for geotechnical engineering. The common type of Fly-ash is generally composed of crystalline compounds such as quartz (SiO_2_), mullite (Al_6_Si_2_O_13_) and silimanite (Al_2_SiO_5_), glassy compounds such as silica glass and other oxides. Fly-ash is a granular material and contains many hazardous components and thus cannot be left untreated in an open environment as it may cause air pollution and respiratory problems to the local inhabitants. Also, when untreated Fly-ash is deposited at the dump-sites and land-fills, it leaches out due to rain water and pollutes the local water bodies thus affecting the flora and fauna of the region [1]. Due to its toxicity and the huge amounts in which it is produced daily, the disposal of Fly-ash is a major problem and requires the wastes to be either immobilized with cement and deposited in controlled landfills or stored in underground repositories, which is both costly and time consuming.

Interestingly, some important metal elements like Si and Al are present in huge concentrations in the Fly-ash and can allow an economic recovery from this waste. Out of the various leaching procedures which can be used, fungal mediated bio-leaching is the most reasonable one as it not only selectively leaches out these metals but also converts them to their nano form which get naturally protein capped during the process. Also, the by-products of this reaction can be used for the synthesis of alumino-silicate compounds like Zeolite which have important applications in catalytic reactions. Thus, the fungal mediated bio-leaching which is an eco-friendly, cheap, non- toxic process occurring at ambient conditions, not only leads to economic recovery from wastes but also significantly reduces pollution and helps protect the environment. From this viewpoint, Fly-ash may indeed be considered as an “artificial ore” [2].

The interaction of microorganisms with metals may be defined as bioleaching, which causes the solubilization of metals and the transformation of solid compounds which ultimately results in the economical recovery of extracted elements [3]. The mobilization and leaching of metals from solid compounds by microorganisms mainly depends on three principles, namely (i) the transformation of organic or inorganic acids (protons); (ii) oxidation and reduction reactions; and (iii) the excretion of complex agents. The continuous discharge of highly polluted effluents in the forms of liquids, solids and gases by the global industrial activities, resulted in the rapid deterioration of the ecosystem. Thus, it has become need of the hour to develop eco-friendly and sustainable processes in order to prevent the rapid degradation of the ecosystem. Hence, concerted efforts are being put to develop eco-friendly processes especially in the fields of metal extraction and mineral processing, which have been the bulk of world economy [4]. Generally, pyro- and hydro-metallurgical routes or a combination of both are being used to recover metals from their respective ores. But due to the gradual depletion of high-grade ores, these high value metals are now recovered from wastes, complex and lean ores which otherwise cannot be treated economically by conventional routes [5], [6]. The reuse of such materials conserves the non-renewable resources as well as solves the problem of environmental deterioration. Several other important inorganic metals such as copper, iron, gold, etc. are produced by organisms via bioleaching at commercial level.

We have already reported the biotransformation of the chemical precursor K_2_SiF_6_ into highly stable crystalline silica nanoparticles at room temperature [7]. In order to make this fungal-mediated nanoparticle synthesis completely biogenic, we replaced the chemical precursors with naturally available materials and agro-industrial by-products. Our group in the past has demonstrated that the fungus *Fusarium oxysporum* could be used for selective bioleaching of crystalline silica nanoparticles from white sand and zircon sand [8], [9]. In another experiment carried out by our group, it has been shown that *F. oxysporum* when exposed to rice husk is not only capable of leaching out huge amounts of amorphous silica present in the rice husk in the form of flat, porous silica nanostructures; but more interestingly, the fungus also biotransforms this amorphous silica into crystalline silica particles at room temperature [10].We in the past have also obtained silicate nanoparticles using the fungus *Humicola* sp. at 50°C by bioleaching of glass with the accompanied modification of the glass surface [11].

Silica nanoparticles have always been a center of attraction for researchers due to their innumerable technological applications and biological importance. Nano silica also plays an important role in silica-based materials such as resins, catalysts and molecular sieves [12]. Silica nanoparticles have started gaining importance in biology and medicine too. Bioconjugated and doped silica nanoparticles in particular are very important in cancer cell imaging [13],ultrasensitive single bacterium detection [14], DNA and microarray detection [15], bar-coding tags [16], separation and purification of biological molecules and cells [17] and gene/drug delivery [18].Owing to all these reasons, ‘bioleaching’ has become a potential tool for eco-friendly, low-cost synthesis of various metals from their precursors. In the present work, we have demonstrated the capability of prefrential leaching of highly crystalline, fluorescent and stable silica nanoparticles out of several other components present in Fly-ash obtained from thermal power plants by the fungus *Fusarium oxysporum*.

## Materials and Methods

Fly-ash was obtained from thermal power plant (Chandrapur, Maharashtra, India), malt extract, yeast extract, glucose and peptone were obtained from HiMedia and used as-received.

### Bioleaching of fly-ash for the production of silica nanoparticles

The mesophilic fungus *Fusarium oxysporum* was isolated from plant material and maintained on MGYP (malt extract, glucose, yeast extract and peptone) agar slants. Stock cultures were maintained by sub culturing at monthly intervals. After growing the fungus for 96 h, the slants were preserved at 15°C. From an actively growing stock culture, subcultures were made on fresh slants and after 96 h of incubation were used as the starting material for fermentation experiments. For the bioleaching of Fly-ash and subsequent production of silica nanoparticles, the fungus was grown in 250 ml Erlenmeyer flasks containing 100 ml of MGYP medium which is composed of malt extract (0.3%), glucose (1%), yeast extract (0.3%) and peptone (0.5%). The culture was grown with continuous shaking on a rotary shaker (200 rpm) at 25°C for 96 h. After 96 h of fermentation, mycelial mass was separated from the culture broth by centrifugation (5000 rpm) at 20°C for 20 min and was then washed thrice with sterile distilled water under sterile conditions. The harvested mycelial mass (60 g of wet mycelia) was then mixed with 10 g of Fly-ash and suspended in 100 ml of distilled water(pH 6.5) in 500 ml Erlenmeyer flask. The whole mixture was put onto a shaker at 25°C (200 rpm) and maintained in the dark.

### Characterization of bioleached silica nanoparticles

#### UV-visible spectroscopy measurement

Aliquots(2 ml) of the bioleached silica nanoparticles were removed at regular intervals and UV-vis spectrophotometric measurements were carried out on a Perkin Elmer dual-beam spectrophotometer (Model lambda 750) operated at a resolution of 1 nm.

#### Photoluminescence (PL) measurements

Aliquots of the reaction mixture were removed at regular intervals and subjected to the fluorescence measurements, which were carried out using a Perkin-Elmer LS 50B luminescence spectrophotometer.

#### Transmission Electron Microscopy (TEM) measurements

The size and shape analysis of bioleached silica nanoparticles was done on a TECHNAI G2 F20 S-TWIN instrument operated at voltage of 200 KV. HR-TEM measurements were carried out on a TECHNAI G2 F30 S-TWIN instrument (Operated at an acceleration voltage of 300 kV with a lattice resolution of 0.14 nm and a point image resolution of 0.20 nm). For TEM and HR-TEM measurements, the samples were prepared by drop-coating the particles suspended in aqueous medium on carbon coated copper grids. Selected area electron diffraction (SAED) analysis was carried-out on the same grids.

#### X - Ray Diffraction pattern (XRD) measurements

XRD patterns were recorded using a PHILIPS X'PERT PRO instrument equipped X'celerator, a fast solid-state detector on drop-coated sample on glass substrate. The sample was scanned using X'celerator with a total number of 121 active channels. Iron-filtered Cu Kα radiation (λ = 1.5406 Å) was used. XRD patterns were recorded in the 2θ range of 20°–80° with a step size of 0.02° and a time of 5 seconds per step at 40 kV voltage and a current of 30 mA.

#### Fourier Transform Infrared Spectroscopy (FTIR)

FTIR spectroscopy measurements on bioleached silica nanoparticles powder taken in KBr pellet were carried out using a Perkin–Elmer Spectrum One instrument at a resolution of 2 cm^−1^.

#### Energy Dispersive Analysis of X-rays (EDAX)

Energy Dispersive Analysis of X-rays (EDAX) measurements of the bioleached silica nanoparticles were carried out on a Leica Stereoscan-440 SEM equipped with a Phoenix EDAX attachment. EDAX spectra were recorded in the spot-profile mode by focusing the electron beam onto a region on the surface coated with bioleached silica nanoparticles.

## Results and Discussion

**Figure 1:**
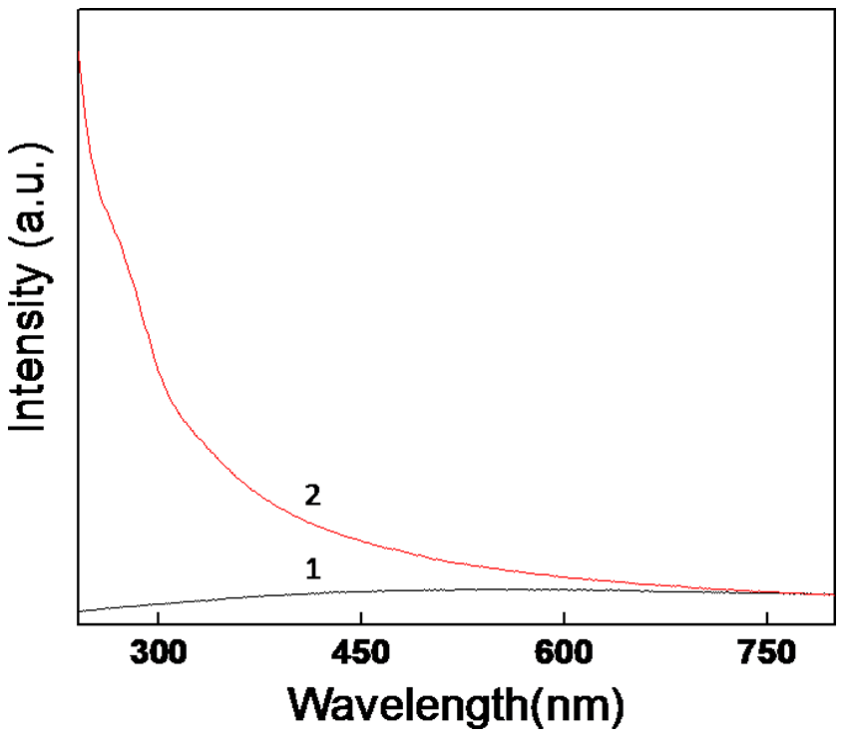
UV-visible spectroscopy measurements of Fly-ash powder curve (1) and the bioleached silica nanoparticles after a reaction of 24 h between the fungus *Fusarium oxysporum* and Fly-ash powder curve (2).

Figure 1 shows the UV-visible spectroscopy measurements of the Fly-ash powder (curve 1) and bioleached silica nanoparticles after a reaction of 24 h between the fungus *Fusarium oxysporum* and Fly-ash. The UV-visible spectrum of Fly-ash powder (curve 1) does not show any absorption in the entire region (scan from 240–800 nm) whereas the UV-visible spectrum of bioleached silica nanoparticles shows a prominent absorption at *ca* 275 nm and a absorption edge at *ca* 350 nm. Absorption at 275 nm can be assigned to the presence of aromatic amino acids such as tryptophan, tyrosine and phenylalanine [19]. These amino acids are present in the proteins and enzymes secreted by the fungus while leaching of Fly-ash, thus confirming the role of certain biomolecules in the bioleaching process. When the fungal biomass reacts with Fly-ash, the specific proteins and enzymes secreted by the fungus in response to stress conditions act predominantly on the silica component of Fly-ash. Moreover, it has already been reported that *Fusarium oxysporum* has more affinity towards silica [9].This interaction of proteins and enzymes results in the formation of an enzyme-silicic acid complex (initial bioleached product) [10]. This siliceous complex will be hydrolyzed by the action of specific hydrolyzing enzymes again secreted by the fungus. The isoelectric point of silica (pI-2) is much lower than that of other components of Fly-ash, which made us to assume that the enzymes/proteins that are acting on siliceous complex are maybe of cationic nature. This siliceous complex which is present within biomass will be then released into the solution in the form of silica nanoparticles. Since Fly-ash comprises mainly of silica, mullite and silimanite (SiO_2_, Al_6_Si_2_O_13_ and Al_2_SiO_5_ respectively), at this point of time we are not sure about which out of these three silica components the enzymes are acting upon. The source of bioleached silica nanoparticles which are released into the solution may be of nascent silica or mullite/silimanite or all the silica constituents present in the Fly-ash. However, a probable mechanism can be derived for the leaching of SiO_2_ from all these components. Fungal metabolism and metabolic enegry involvement during the reaction between *Fusarium oxysporum* and the silica component of the Fly-ash might be playing a role which leads to not only the extracellular leaching of crystalline silica as it is into the solution, but also in nanoregime due to natural protein capping. The leaching of crystalline silica from mullite (Al_6_Si_2_O_13_) and silimanite (Al_2_SiO_5_) may be explained by following reactions: 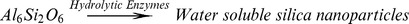


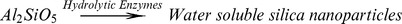



**Figure 2:**
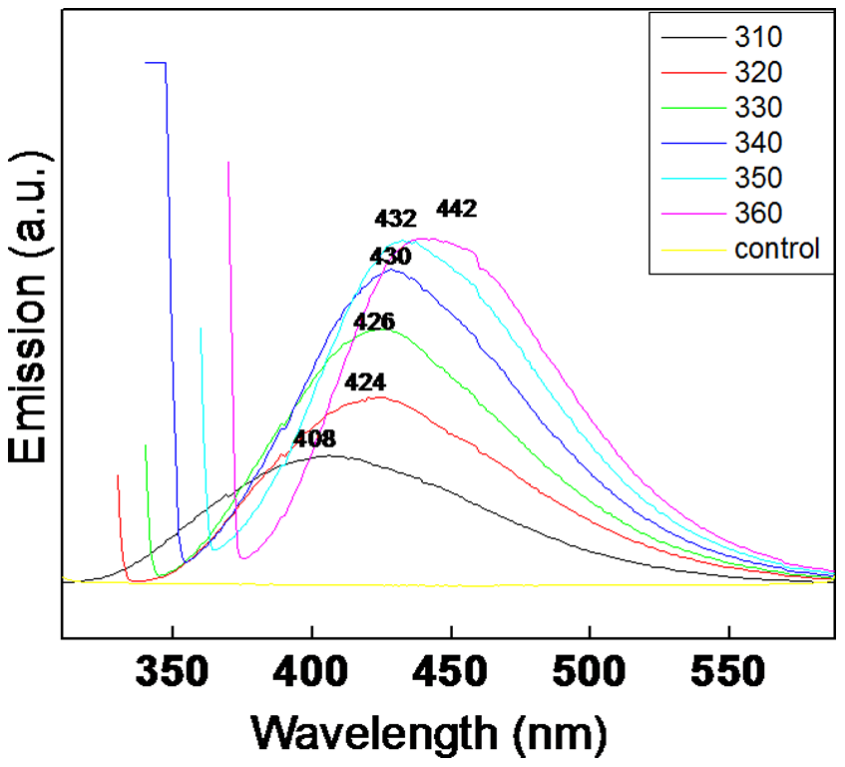
Photoluminescence spectrum of Fly-ash powder and bioleached silica nanoparticles (excited at different wavelengths) obtained after 24 h reaction between *Fusarium oxysporum* and Fly-ash.

**Figure 3:**
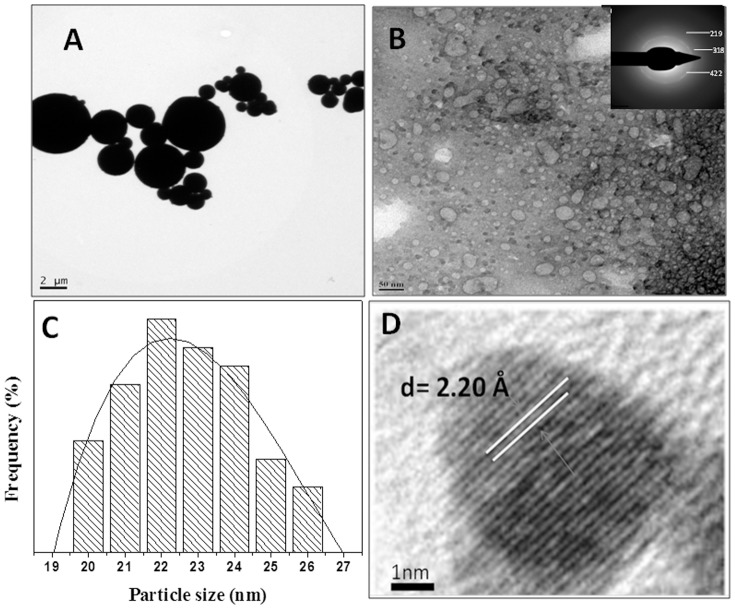
Transmission Electron Microscopic image of Fly-ash powder(A), bioleached silica nanoparticles obtained after reacting Fly-ash with *Fusarium oxysporum* for 24 h(B) inset shows selected area electron diffraction (SAED) analysis of bioleached silica nanoparticles, particle size distribution analysis of bioleached silica nanoparticles(C) and HR-TEM image of bioleached silica nanoparticles (D).

**Figure 4:**
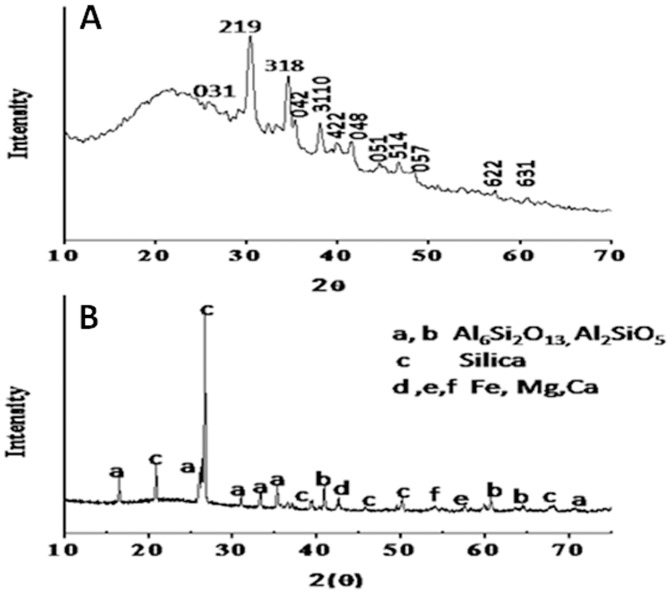
X-ray Diffraction (XRD) analysis of bioleached silica nanoparticles obtained after reacting Fly-ash with *Fusarium oxysporum* for 24 h (A) and Fly-ash powder (B).

**Figure 5:**
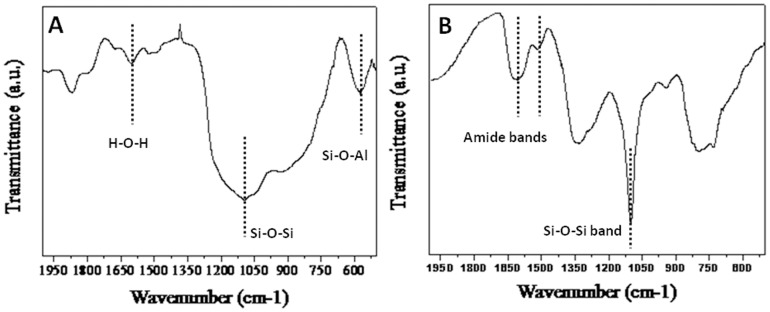
Fourier Transform Infrared (FTIR) spectrum of Fly-ash powder (A) and bioleached silica nanoparticles obtained after reacting Fly-ash with *Fusarium oxysporum* for 24 h (B).

**Figure 6:**
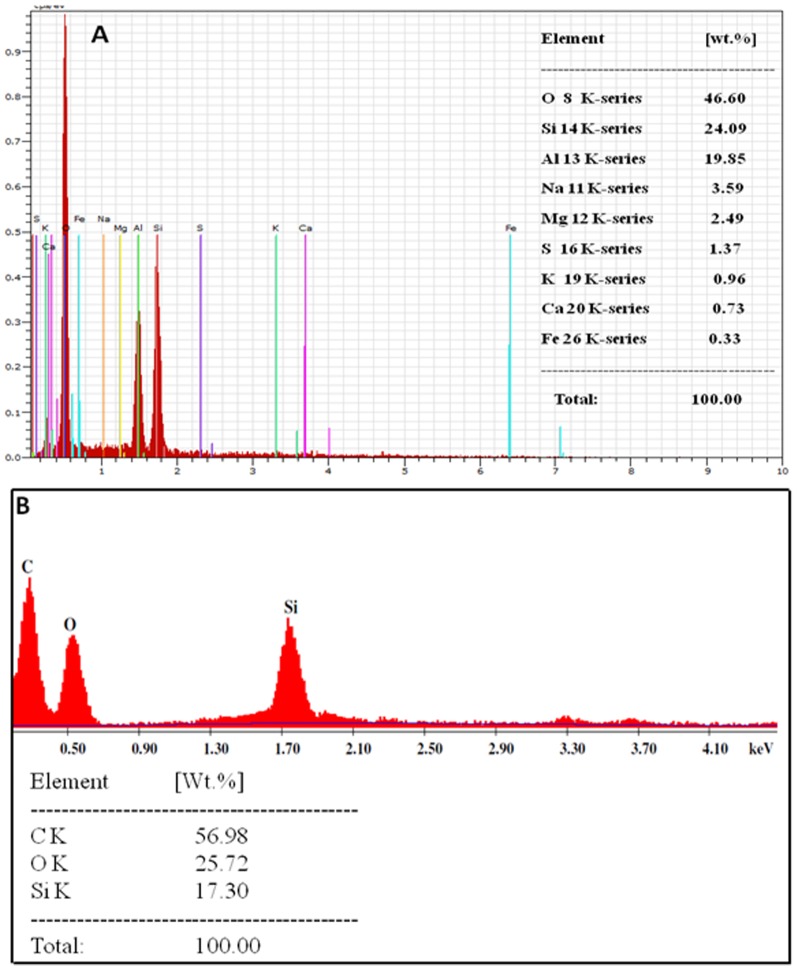
Energy Dispersive Analysis of X-rays (EDAX) spectrum and wieght percentages of Fly-ash powder(A) and bioleached silica nanoparticles obtained after reacting Fly-ash with *Fusarium oxysporum* for 24 h(B).

In the above reactions, enzymes of fungus are acting on mullite (Al_6_Si_2_O_13_) and silimanite (Al_2_SiO_5_) to form siliceous-enzyme complex which is then acted upon by hydrolyzing enzymes of fungus finally resulting into silica and alumina, out of which silica comes out of the biomass and is released into the solution within 24 hr of reaction. The absorption edge at *ca* 350 nm thus can be attributed to the bioleached silica nanoparticles which are released into the solution extracellularly. In order to negate the possibility of leaching of silica nanoparticles from fly-ash due to acidic nature of the reaction medium, a control experiment (Figure S1) was performed by keeping the fly-ash in distilled water in the absence of *Fusarium oxysporum* and maintained at pH-4 for 24 h. The filtrate was then characterized by UV-Vis, XRD, TEM and photoluminescence. Figure (S1) shows the UV-Vis, XRD, TEM and photoluminescence analyses of the filtrate of the solution in the absence of *Fusarium oxysporum.* From the figure(S1) it is very clear that no silica or protein peak is coming in the UV-Vis spectroscopy and XRD measurements nor any particle could be seen under TEM analysis and photoluminescence also was not detected. Hence it is quite obvious that the leaching of silica nanoparticles from fly-ash is carried out by the fungus *Fusarium oxysporum.* Figure 2 shows the photoluminescence (PL) spectrum of Fly-ash powder (control) and bioleached silica nanoparticles after reaction of 24 h between the fungus *Fusarium oxysporum* and Fly-ash. When the bioleached silica nanoparticles exicted at different wavelengths such as 310, 320, 330, 340, 350 and 360 nm, gave emission bands at 408, 424, 426, 430, 432 and 442 nm respectively whereas Fly-ash powder did not give any emission bands when excited at same wavelengths. This phenomenon of photoluminescence occurs when there is interface between Si nanocrystals in SiO_2_ matrix at nanoscale size regime [20]. To the best of our knowledge this is the first report on photoluminescence of biotransformed silica nanoparticles which can be useful in biomedical applications such as diagnostics, biosensors, drug delivery etc. Figure 3(A) shows a representative Transmission Electron Microscopic image of Fly-ash powder (control) showing very big particles ranging in few micrometers. Figure 3(B) shows a representative TEM image of bioleached silica nanoparticles obtained after reacting Fly-ash with *Fusarium oxysporum* for 24 h.The particles are polydispersed and quasi-spherical in shape. These nanoparticles are capped by capping biomolecules and makes them water dispersible. Inset in figure 3(B) shows Selected Area Electron Diffraction (SAED) analysis of bioleached silica nanoparticles. The diffraction spots in the SAED pattern could be well indexed based on the silica structure [21]. Figure 3(C) represents a particle size distribution histogram of bioleached silica nanoparticles showing that the particles are in the range of 20–26 nm with an average diameter of 22 nm. HR-TEM analysis of bioleached silica nanoparticles (figure 3D) revealed the inter planar distance to be 2.20 Å which corresponds to the plane {048} of SiO_2_. The TEM and SAED results clearly show that crustalline silica nanoparticles are leached out from Fly-ash by the fungus *Fusarium oxysporum.* Figure 4 represents the X-ray diffraction analysis of (A) bioleached silica nanoparticles obtained after reacting Fly-ash with *Fusarium oxysporum* for 24 h and (B) Fly-ash powder (control). The XRD pattern of bioleached silica nanoparticles showed peaks at {031},{219}, {318}, {042},{3110}, {422}, {048}, {051}, {514}, {057}, {622} and {631} in the 2θ range of 20–80° and agrees well with crystalline polymorph of silica [21]. The broadened XRD peaks reflect the small size of silica nanoparticles. It is important to note here that even though the particles are capped by proteins, presence of proteins does not compromise with the crystallinity of the bioleached silica nanoparticles which can be confirmed by the occurrence of sharp Bragg reflections even without calcinations of as-bioleached silica particles. The X-ray diffraction analysis of Fly-ash powder showed different peaks corresponding to different components of Fly-ash (such as Al_6_Si_2_O_13_, Al_2_SiO_5_, Si, Fe, Mg and Ca). Figure 5(A) represents the Fourier transform infrared (FTIR) spectrum of Fly-ash powder (control) that shows the presence of different vibrations corresponding to Fly-ash components. The peak at 600 cm^−1^ corresponds to Si-O-Al streching vibration [22] whereas peaks at 1098 and 1608 cm^−1^ may be attributed to Si-O-Si asymmetric strecthing vibration [23] and H-O-H bending vibration [24] respectively. Fly-ash spectrum does not contain any peak which corresponds to amide region. This confirms that SiO_2_ nanoparticles capped with proteins releases into the solution extracellularly only when *Fusarium oxysporum* reacts with Fly-ash powder for 24 h. Figure 5(B) represents the FTIR analysis of bioleached silica nanoparticles obtained after reacting Fly-ash with *Fusarium oxysporum* for 24 h. The FTIR spectrum shows a sharp peak at *ca* 1098 cm^−1^. This peak can be assigned to the Si–O–Si antisymmetric stretching mode of silica [23]. Thus; it is made very clear that the silica nanoparticles are leached out by the fungus in the extracellular solution. Figure 5(B) also shows the presence of two peaks at *ca* 1641 and 1540 cm^−1^ which can be assigned to amide I and amide II bands respectively. These amide bands may emanate from the proteins during the reaction with Fly-ash which are responsible for capping of bioloeached silica nanoparticles. Figure 6 represents the Energy Dispersive Analysis of X-rays (EDAX) spectrum and weight percentage of (A) Fly-ash powder (control) and (B) bioleached silica nanoparticles obtained after reacting Fly-ash with *Fusarium oxysporum* for 24 h. The spectrum of Fly-ash powder shows the presence of different constittuents such as Si, Al, Fe, Mg, Ca etc. whereas the EDAX spectrum of bioleached silica nanoparticles shows peaks at 1.74 KeV and at 0.51 KeV corresponding to Si and O respectively. The presence of Si and O in the bioleached sample confirms the presence of silica nanoparticles. These results are in good agreement with XRD and FTIR analysis (figure 4&5B) confirming the bioleaching of Fly-ash by the fungus accompanied by the predominant release of silica nanoparticles in the solution (filtrate). As can be evident from both the figures the percentages of silica were 24.09 and 17.30 before and after the reaction with the fungus *Fusarium oxysporum* respectively.

Now, Total % of Silica nanoparticles in solution is given by 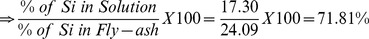



Hence 71.81% of silica present in the fly-ash is converted into silica nanoparticles or leached out from the fly-ash.

We for the first time have shown that the fungus *Fusarium oxysporum* when exposed to Fly-ash is able to leach highly crystalline fluorescent silica nanoparticles extracellulary in solution at room temperature. Hence, a cheap and environment friendly approach has been derived to obtain commercially important silica nanoparticles out of waste materials using a fungus, thus negating the requirement of any chemical precursors and making the process purely biogenic. Thus, this process not only leads to economic recovery but also helps in waste management and pollution control. The reaction conditions and parameters involved in the above bioleaching process can be fine tuned in order to obtain porous or hollow silica, which could prove very useful by virtue of its room temperature photoluminescence and high drug loading capacity inside its pores for biomedical applications.

## Conclusions

In conclusion, we have shown the ability of the mesophilic fungus *Fusarium oxysporum* in the bioleaching of waste material such as Fly-ash for the extracellular production of highly crystalline and highly stable, protein capped, fluorescent and water soluble silica nanoparticles at ambient conditions. We believe that this non-hazardous approach can also be extended towards extracting technologically challenging nanomaterials from other minerals such as illmenite, chromite, magnetite, bauxite, etc. which are present in large quantities in natural environments.

## Supporting Information

Figure S1 **UV-vis spectroscopy(A), XRD(B) measurements, TEM(C) and PL(D) analyses of filtrate in the absence of** ***Fusarium oxysporum*** **.**
(TIFF)
