# Supplementary figures and images for: Fungus-Mediated Preferential Bioleaching of Waste Material Such as Fly - Ash as a Means of Producing Extracellular, Protein Capped, Fluorescent and Water Soluble Silica Nanoparticles
Source: PLoS One. 2014 Sep 22;9(9):e107597. doi: 10.1371/journal.pone.0107597 (PMC4171486; doi:10.1371/journal.pone.0107597)

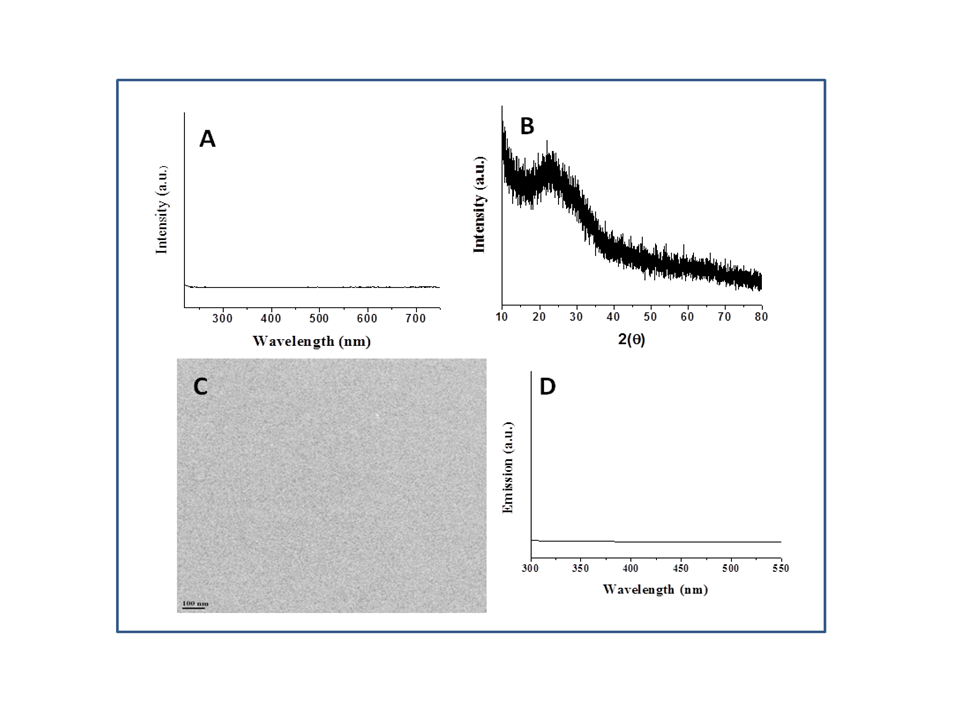

Supplement: Figure S1 — UV-vis spectroscopy(A), XRD(B) measurements, TEM(C) and PL(D) analyses of filtrate in the absence of Fusarium oxysporum . (TIFF) [file pone.0107597.s001.tiff]
